# Yellow Teas Protect against DSS-Induced Ulcerative Colitis by Inhibiting TLR4/NF-κB/NLRP3 Inflammasome in Mice

**DOI:** 10.3390/foods13172843

**Published:** 2024-09-07

**Authors:** Dawei Xing, Tao Zheng, Xiaoju Chen, Zhongwen Xie

**Affiliations:** 1 Chaohu Regional Collaborative Technology Service Center for Rural Revitalization, School of Biological and Environmental Engineering, Chaohu University, 1 Bantang Road, Hefei 238024, China; xingdw95@126.com (D.X.); taozheng030@outlook.com (T.Z.); 2 State Key Laboratory of Tea Plant Biology and Utilization, Anhui Agricultural University, 130 Changjiang West Road, Hefei 230036, China; 3 Joint Research Center for Food Nutrition and Health of IHM, Anhui Agricultural University, 130 Changjiang West Road, Hefei 230036, China

**Keywords:** yellow tea, ulcerative colitis, NLRP3 inflammasome

## Abstract

Yellow tea (YT), a slightly fermented tea with a unique yellowing process and mellow taste, is becoming widely popular. Currently, the YT includes bud yellow tea (BYT), small-leaf yellow tea (SYT), and large-leaf yellow tea (LYT) based on maturity of raw materials. Previous studies have shown that YT has outstanding potential in preventing metabolic syndrome. However, the distinct effects and mechanisms of different types of YT on ulcerative colitis (UC) are still unclear. This study investigated the effects and mechanisms of continuous or intermittent intervention of three yellow tea water extracts (YTEs) on dextran sulfate sodium (DSS)-induced ulcerative colitis in CD-1 mice. The results showed that YTE intervention significantly improves the syndrome of DSS-induced UC in mice. Mechanistic studies reveal that YTEs increase the expression levels of tight junction (TJ) proteins and reduce the levels of pro-inflammatory cytokines in the colon by inactivating TLR4/NF-κB/NLRP3. YTE treatment protected intestinal barrier integrity and reduced serum lipopolysaccharide (LPS) levels. Interestingly, our results indicate that large-leaf yellow tea (LYT) has a better alleviating effect than BYT and SYT. YTE intervention before DSS administration has a certain degree of preventive effect on ulcerative colitis, while continuous YTE intervention after DSS induction has a significant reversing effect on the damage caused by DSS. Our results indicated that drinking YT may have preventive and therapeutic effect on UC, especially drinking LYT.

## 1. Introduction

The incidence and prevalence of inflammatory bowel disease (IBD) has rapidly increased worldwide, spreading in some newly industrialized countries in Asia, South America, and the Middle East [1]. As one of the main types of IBD, ulcerative colitis (UC), a persistent diffuse chronic inflammatory bowel disease originating in the rectum and extending to the proximal colon [2], has been reported to be caused by genetic background, environmental factors, dietary habits, and immune dysfunction [3]. Although the occurrence and development of UC are closely related to intestinal oxidative stress, mucosal immunity, inflammatory factors, changes in intestinal flora, etc. [4,5,6], its specific mechanisms have not been fully elucidated. Meanwhile, although the current treatment for UC mainly focuses on medication, surgery, and stress management [7], these existing treatment methods cannot completely cure UC. While biological drugs bring side effects to the human body, their high cost also puts enormous pressure on the medical system. Therefore, finding a diet or beverage that can intervene and prevent UC has important theoretical and application value.

The pathological characteristics of UC are mucosal epithelial damage and intestinal homeostasis disruption, which allows commensal and pathogenic bacteria to penetrate the epithelium and cause an inflammatory response, leading to leukocyte recruitment and dysregulated intestinal immune response [8]. Epithelial cells are connected by tight junctions (TJs), forming a tight barrier that prevents microorganisms in the lumen from triggering an immune response, and the expression levels of transmembrane proteins (Occludin, Claudin-1, Claudin-4), peripheral membrane proteins (ZO-1, ZO-2) and binding adhesion molecules (JAM) are closely related to this process [9]. Disruption of the epithelial barrier leads to interactions between gut microbes and immune cells, leading to a progressive out-of-control inflammatory response. Harmful substances represented by lipopolysaccharide (LPS), D-lactic acid (D-LA) and diamine oxidase (DAO) penetrate the mucosa and enter the circulation and activate the corresponding NF-κB and mitogen-activated protein kinase (MAPK) pathways, ultimately causing the release of IL-1, IL-6, TNF-α, NO, etc., inhibiting the expression of IL-10, and forming an inflammatory response [10].The innate immune system is an important defense mechanism that allows the body to fight off microorganisms in a non-specific manner. As an important component of the innate immune system, the NLRP3 inflammasome has been intensively studied. The NLRP3 inflammasome is a multiprotein complex composed of a nucleotide-binding domain and leucine-rich repeat protein (NLR), the bridging protein ASC, and caspase-1 [11]. After recognizing pathogen-associated molecular patterns or damage-associated molecular patterns, the NLRP3 inflammasome is assembled and subsequently releases IL-1β, a pro-inflammatory cytokine, which plays an important role in the pathogenesis of intestinal inflammation [12]. In addition, previous studies have shown that downregulating NLRP3 expression can improve intestinal inflammation, and NLRP3 knockout (KO) mice are protected from DSS-induced colitis [13]. Therefore, activation of NLRP3 in the inflammasome is critical in the development of colonic inflammatory processes, suggesting that functional compounds inhibiting NLRP3 inflammasome activation may have potential to treat inflammatory diseases.

Yellow tea (YT), one of the six major tea categories, originated from the Ming Dynasty and has a unique yellowing process [14]. YT can be divided into three main types: bud yellow tea (BYT), small-leaf yellow tea (SYT), and large-leaf yellow tea (LYT), according to the maturity of different raw tea shoots. BYT is made from buds, or one bud and one newly grown leaf, SYT is made from one bud and one or two newly grown leaves. However, LYT is manufactured from shoots, including 3 to 5 mature tea leaves [15]. YT, especially LYT, shows outstanding potential in preventing obesity [16], hepatic steatosis [17], hyperuricemia [18], and hyperglycemia [19]. The modulating effect of YT on the gut microbiota also mitigates IBD, metabolic syndrome and gastrointestinal diseases [20,21,22]. The research also showed that YT reduces hyperglycemia-induced vascular and kidney damage, and alcohol-induced liver and gastric damage [23,24]. However, the distinct effects and mechanism of the different types of YT on IBD have not been comparatively investigated. In addition, previous studies also pay little attention to comparing the preventive and therapeutic effects of YT on IBD. In this study, the mechanism and preventive and alleviating effects of three YTs on DSS-induced ulcerative colitis will be comparatively investigated.

## 2. Materials and Methods

### 2.1. Chemicals and Reagents

DSS (MW, 36,000–50,000 Da) was purchased from MP Biomedicals (LLC, Santa Ana, CA, USA); Lab mice diet (Fat12%, Carbohydrate 67.4%, Protein 20.6%, 3.5 kcal/g) was purchased from Xietong Pharmaceutical Bio-engineering Co., Ltd. (Nanjing, China).

### 2.2. Preparation of YT Water Extracts

LYT, SYT and BYT samples were obtained from Bao Er Zhong Xiu Tea Industry Co., Ltd. (Huoshan, Anhui, China) and were extracted according to a previously reported method [17]. Briefly, 200 g of dried tea samples of LYT, SYT, and BYT was added to the crusher to obtain tea powder, respectively. The powder was passed through an 80-mesh sieve to obtain around 140 g of fine tea powders, which was extracted three times by boiled water with ultrasound assistance. The ultrasound-assisted extraction was performed with power set at 50 W and at 75 °C for 40 min. Afterwards, the extractions were concentrated under vacuum and lyophilized, yielding YT water extracts (YTE) which were stored at −80 °C for subsequent use.

### 2.3. Animal Experimental Design

Six-week-old specific pathogen-free (SPF) CD-1 male mice (32–36 g) were obtained from Vital River Laboratories (Beijing, China). The mice were free to drink and eat and were kept in the specific pathogen-free laboratory animal center (temperature 22 ± 1 °C, relative humidity 50 ± 5% with 12 h light-dark cycle) at Anhui Agricultural University.

**Figure 1:**
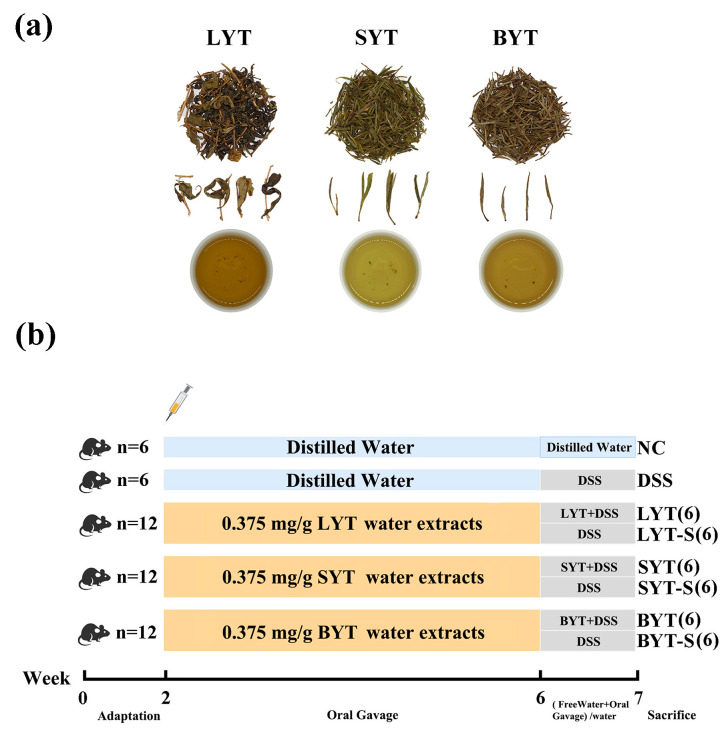
Experimental design. (**a**) Dry samples and water infusion of three YTs used in this study; (**b**) schematic of experimental design.

The mice were acclimatized for two weeks and randomly divided into five groups, including control group (NC) (n = 6), DSS group (DSS) (n = 6), LYT intervention group (LYT) (n = 12), SYT intervention group (SYT) (n = 12), BYT intervention group (BYT) (n = 12). The mice in the LYT group, SYT group and BYT group were given 0.375 mg/g of the corresponding YTE (Figure 1a) by oral gavages once a day for a total of 4 weeks. During the following week, colitis was induced by administering 2% (*w*/*v*) DSS via drinking water, while the NC group was given the same amount of water. One week after DSS induction, half of the mice in the LYT group, SYT group and BYT group continued to be treated with 0.375 mg/g of the corresponding YTE (n = 6), while the other half of mice drank the same amount of distilled water and were renamed the LYT-S group (n = 6), SYT-S group (n = 6) and BYT-S group (n = 6) (Figure 1b). In all animal experiments, YTE was prepared freshly every day just before administration. All mice experiments were approved by the Institutional Animal Care and Use Committee of the Anhui Agricultural University (ethical code: AHAU 20200024).

### 2.4. Samples Collection

The overnight-fasting mice were euthanized under anesthesia at the end of each treatment. Whole blood was collected and kept at room temperature for 15 min without being disturbed. The serum was prepared by centrifuging for 10 min at 3000× *g* at room temperature, and then frozen at −80 °C until use. After dissection, a colon sample was rapidly collected, and its length was measured. And a small piece of colon was cut at the same position across various group of mice and was fixed in 10% buffered formalin. The other colon tissue was frozen in liquid nitrogen and stored at −80 °C for further analysis.

### 2.5. Evaluation of the Disease Activity Index (DAI)

The mice weight, stool consistency and fecal occult blood status were recorded daily. The DAI score was given for the three parameters based on scoring criteria to calculate the disease activity index of each mouse. The scoring criteria refer to previous studies [25], and the DAI is defined as shown in Table S1.

### 2.6. Histological Analysis

Histological analysis was conducted following a previously described method [26]. Briefly, the colon tissue in paraffin was cut into 5 µm sections. The colon sections were stained with an H&E staining kit (Boster Biotech, Wuhan, China). The pictures were captured using a microscope (LEICA DM500, Wetzlar, Germany) with a camera (LEICA ICC50 W). Histological scoring was performed using methods described in previous studies [27]. The details of histological scoring are shown in Table S2.

### 2.7. Immunofluorescence of Colon Tissue

The ZO-1 and Claudin-1 expression was detected on the paraffin section of colon tissue using immunostaining following a previously reported method [17]. Briefly, the sections were deparaffinized, rehydrated and washed with PBS, were blocked with 5% BSA for 30 min at room temperature, and were further incubated with anti-ZO-1 and anti-Claudin-1 antibodies (1:100) for 1 h. The slides were treated with DAPI (1:500) for 30 min under dark conditions. Finally, images were captured using a Leica SP8 microscope (LEICA, Wetzlar, Germany). Mean fluorescence intensity was measured and statistically analyzed using Leica LAX software (LAS_X_4.7.0_28176).

### 2.8. Determination of the Serum Cytokine Concentration

The concentration of LPS, TNF-α, IL-1β and IL-6 in serum was determined by using enzyme-linked immunosorbent assay (ELISA) kits (Jianglai Biotech, Shanghai, China). The amount of LPS, TNF-α, IL-1β and IL-6 in the serum was quantified according to the manufacturer’s instructions.

### 2.9. Quantitative Real-Time PCR Analysis

RNA-easy Isolation Reagent (Vazyme, Nanjing, China) and HiScript II cDNA Synthesis kit (Vazyme, Nanjing, China) were used to extract RNA and synthesize cDNA, respectively. AceQ qPCR SYBR Green kit (Vazyme, Nanjing, China) was used to conduct quantitative real-time PCR (qRT-PCR), and the results were calculated using the 2^−ΔΔCt^ method to calculate relative expression of target genes. The qRT-PCR system and procedures refer to a previous study [28], and all primers used for qRT-PCR analysis are listed in Table S3.

### 2.10. Western Blot Analysis

Western blots were carried out according to a procedure from a previous study [29]. NOD-like receptor thermal protein domain-associated protein 3 (NLRP3), phospho-p65 (p-p65), total p65, phospho-IκBα (p-IκBα), and total IκBα antibodies were from Cell Signaling Technology (Danvers, MA, USA). Caspase-1 and apoptosis-associated speck-like protein containing CARD (ASC) antibodies were from Santa Cruz Biotechnology (Santa Cruz, CA, USA). β-actin antibody was from Protein tech (Chicago, IL, USA). Bio-Rad ChemicDoc MP Imaging System (Hercules, CA, USA) was used to detect the protein bands with ECL reagent (Vazyme, Nanjing, China). 

### 2.11. Statistical Analysis

Graph Pad Prism 9 software was used for statistical analysis. Data are presented as mean ± SEM. ANOVA with Tukey’s test was used throughout the study to compare the multiple groups. Student’s *t*-test was used for screening of colon classes or species differences between the two groups. The *p* ˂ 0.05 was considered significantly different.

## 3. Results

### 3.1. YTs Relieved the Colitis Symptoms in DSS-Induced Colitis Mice

**Figure 2:**
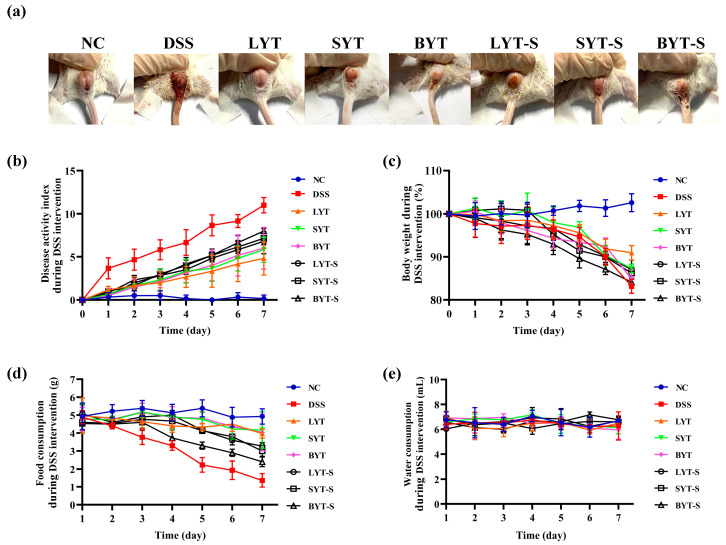
Effects of YTs on the symptom of DSS-induced ulcerative colitis. (**a**) Hematochezia of mices; (**b**) disease activity index; (**c**) body weight; (**d**) food consumption; (**e**) water consumption across various group of mice (n = 6).

To evaluate the anti-colitis effect of YTs, DSS-induced colitis mice model was established to mimic human UC and to determine the preventive and therapeutic effect of LYT, SYT and BYT. The continuous administration of DSS for one week caused diarrhea and hematochezia in the mice (Figure 2a), thus causing the DAI scores to increase continuously (Figure 2b). Pretreatment of all three YTs obviously prevented the syndrome of diarrhea and hematochezia. On the 7th day of administration, the DAI score of mice in the colitis model group was 11, while that of the NC group was 0.17. All treatments with YTs decreased the DAI score. Meanwhile, DSS stimulation led to a gradual decrease in the mice’s body weight (Figure 2c) and food intake (Figure 2d). YT administration prevented body weight loss (Figure 2c) and food intake decrease (Figure 2d). However, there was no significant change in the water consumption of each group during this period (Figure 2e). These results show that pretreatment of YTs for 4 weeks before DSS administration can prevent symptoms caused by DSS to a certain extent. However, continuous YT treatment at the same time of DSS induction for one week showed a better relieving effect than that of stopping YT treatment. Interestingly, among these three YT treatments, LYT had the best prevention and alleviation effects compared to SYT and BYT. There is no significant difference between the SYT and BYT treatments.

### 3.2. YTs Prevented the Epithelial Barrier Damage in the DSS-Induced Colitis Mice

**Figure 3:**
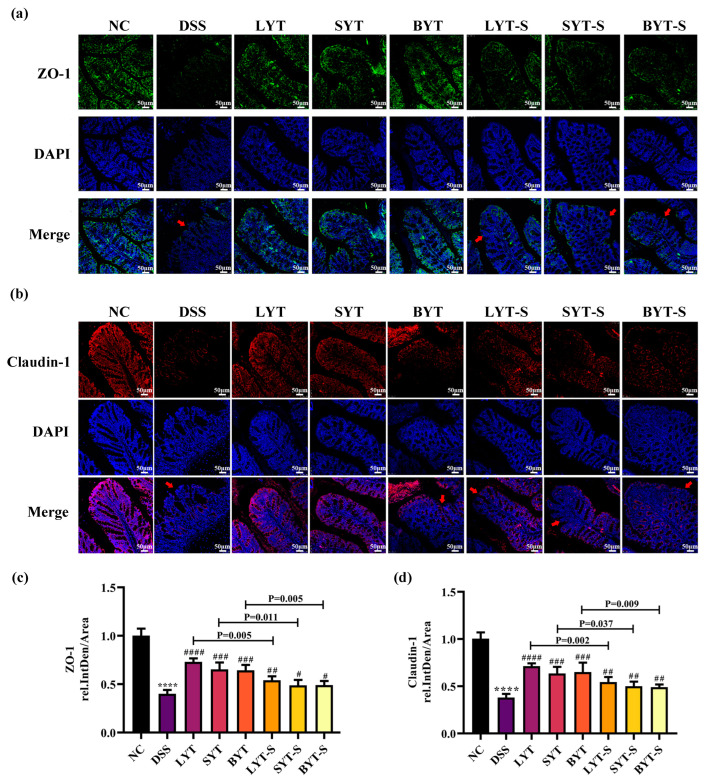
Effects of YTs on TJ protein expression in colon of DSS-induced ulcerative colitis in mice. (**a**) Representative immunofluorescence images of ZO-1 in colon sections (magnification of 100×, scale bar:50 μm); (**b**) representative immunofluorescence staining of claudin-1 in colon section (magnification of 100×, scale bar:50 μm); arrows indicate loss of TJ protein fluorescence; (**c**,**d**) Quantitative data of a (ZO-1) and b (claudin-1), respectively; **** *p* < 0.0001 when compared to NC group; # *p* < 0.05, ## *p* < 0.01, ### *p* < 0.001, #### *p* < 0.0001 when compared to DSS group. *p* value on the bars represents the significant analysis between continued and stopped administration of YT groups (n = 6, data are the means ± SEM).

Tight junction (TJ) proteins of mice colon epithelial cells play an important role in colon integrity [30]. Therefore, we focused on the effect of YTs on the regulation of tight junction proteins of colon epithelial cells in mice with DSS-induced UC. Immunofluorescence staining results showed that DSS treatment led to a dramatic decrease in the expression of TJ proteins ZO-1 and claudin-1, which was almost half that of the NC group mice. Pretreatment of YTs for 4 weeks before DSS induction significantly prevented a decrease in ZO-1 and claudin-1. Continuing to treat the mice with YTs for one week during DSS induction was more efficient to prevent the TJ protein decrease than in the case of stopping the treatment group (Figure 3a,b). In addition, the preventive and therapeutic effects of LYT are significantly better than those of SYT and BYT on ZO-1 and claudin-1 protein decrease. The calculation of fluorescence intensity further confirms these results (Figure 3c,d).

### 3.3. YTs Alleviated Colonic Length Shortening and Epithelial Damage in DSS-Induced Colitis Mice

**Figure 4:**
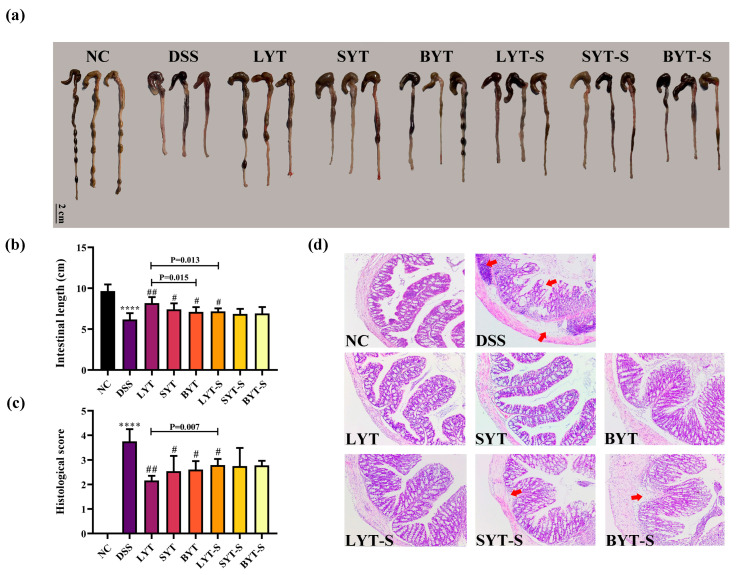
Colonic pathological changes in DSS-induced UC in mice. (**a**) Representative images of colonic in each group (n = 3); (**b**) statistical analysis of colonic length; (**c**) histopathological score; (**d**) H&E staining of colons across various group of mice. Arrows indicate disruption of intestinal epithelial integrity; **** *p* < 0.0001 when compared to NC; # *p* < 0.05, ## *p* < 0.01 when compared to DSS. *p* value on the bars represents the significant analysis between continued and stopped administration of YT groups (n = 6, data are the means ± SEM).

To further confirm the role of YTs in reducing colon damage, we measured the colon length, performed H&E staining analysis, and calculated the histopathological score across various group of mice. The results show that DSS induction resulted in a significant reduction in colon length in mice compared with the NC group, and the three YT administrations significantly alleviated this reduction. Furthermore, LYT had the best effect on preventing colon shortness, followed by SYT and BYT. Stopping LYT treatment during DSS induction had a significantly negative impact on the improvement in colon length shortening. However, there was no significant difference in the colon length of stopping SYT and BYT treatment group compared with the DSS induction group. (Figure 4a,c).

Histopathological examination of the colon section shows that the colon tissue structure of the mice in the NC group was clear and integrated, and there was no congestion, edema, ulcer formation, or inflammatory cell infiltration in the colon mucosa. However, the structural integrity of the colon mucosa of the mice was severely damaged, glands were obviously missing, the intestinal epithelium was also damaged, and a large number of inflammatory cells infiltrated in the DSS-induced group compared to the NC group. Interestingly, all three YT interventions improved the symptoms of colon mucosal injury (Figure 4b). Among them, the LYT showed a better improving effect than that of SYT and BYT. Overall, the relieving effect of continuing the YT treatments was better than stopping the YT treatments at the same duration of DSS induction. These results were further confirmed by histological scoring (Figure 4d).

### 3.4. YTs Suppressed Inflammation in DSS-Induced Colitis Mice

**Figure 5:**
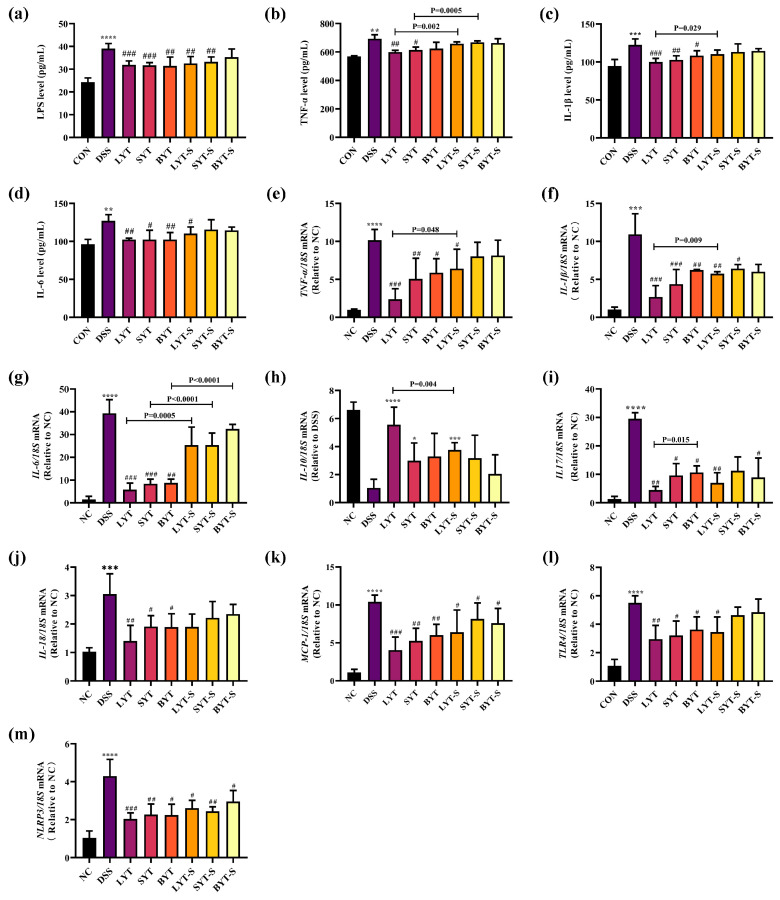
Effect of different types of YT on LPS and cytokines levels in DSS-induced ulcerative colitis mice. (**a**) LPS level; (**b**) TNF-α level; (**c**) IL-1β level; (**d**) IL-6 level in serum, respectively; (**e**) *TNF-α*; (**f**) *IL-1β*; (**g**) *IL-6*; (**h**) *IL-10*; (**i**) *IL-17*; (**j**) *IL-18*; (**k**) *MCP-1*; (**l**) *TLR4*; (**m**) *NLRP3* expression level in the colon across various groups of mice, respectively. * *p* < 0.05, ** *p* < 0.01, *** *p* < 0.001, **** *p* < 0.0001 when compared to NC; # *p* < 0.05, ## *p* < 0.01, ### *p* < 0.001 compared to DSS. *p* value on the bars represents the significant analysis between continued and stopped administration of YT groups (n = 4–6, data are the means ± SEM).

Inflammation plays a pivotal role in the development of ulcerative colitis. First, we explored the expression levels of LPS, TNF-α, IL-1β, and IL-6 in the serum of various groups of mice. ELISA results showed that DSS caused a significant increase in LPS, TNF-α, IL-1β, and IL-6 levels in the serum of DSS-induced mice. YT treatments significantly inhibited this increase (Figure 5a–d). In addition, stopping YT treatments during DSS induction, only LYT still significantly inhibited the expression of LPS and IL-6, and BYT and SYT did not show those alleviating effects. 

Meanwhile, we further evaluated the effect of YTs on the mRNA expression levels of TNF-α, IL-1β, IL-6 and other inflammatory cytokines in the colon of DSS-induced UC mice by qRT-PCR. Consistent with the high LPS load in serum, the expression level of inflammatory cytokines TNF-α, IL-1β, and IL-6 significantly increased in the colons of mice with DSS-induced colitis (Figure 5e–m). Regardless of whether the treatment was continued or stopped during DSS induction, LYT treatment reduced the expression of these inflammatory cytokines to varying degrees. However, after stopping SYT and BYT treatment during DSS induction, only the *MCP-1* and *NLRP3* expression levels were significantly reduced compared to the DSS-induced group (Figure 5k,m).

### 3.5. YTs Inhibited NF-κB/NLRP3 Inflammasome in DSS-Induced Colitis Mice

**Figure 6:**
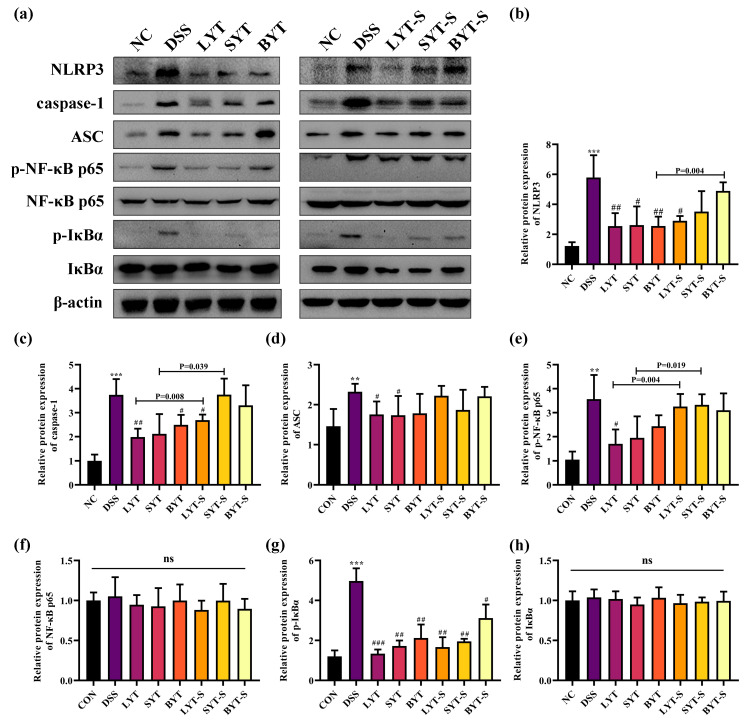
Effects of YTs on NF-κB/ NLRP3 pathway in DSS-induced ulcerative colitis mice. (**a**) The protein levels of NF-κB /NLRP3 inflammasome-associated proteins (NLRP3, caspase-1, ASC, p-NF-κB p65, NF-κB p65, p-IκBα, IκBα) were detected by Western blot; (**b**–**h**) statistical analysis of NLRP3, ASC, p-NF-κB p65, NF-κB p65, p-IκBα, IκBα expression levels, respectively. ** *p* < 0.01, *** *p* < 0.001 when compared to NC; # *p* < 0.05, ## *p* < 0.01, ### *p* < 0.001 when compared to DSS. *p* value on the bars represents the significant analysis between continued and stopped administration of YT groups (n = 4–6, data are the means ± SEM), ns indicates that there is no significant difference between the groups.

The inflammasome is a key research object in the field of immune-inflammatory diseases, and NLRP3 also plays an important role in chemically induced intestinal spontaneous inflammation. Our results showed that high levels of IL-1β are found in DSS-induced UC, which is mainly driven by the NLRP3 inflammasome signaling pathway. To investigate whether the inhibition of IL-1β is due to disruption of NLRP3 signaling, we further analyzed the effect of YTs on the protein expression levels of NLRP3, Caspase-1 and ASC in the colon of DSS-induced UC mice through Western blot (WB) assay. As shown in Figure 6a–d, NLRP3, ASC and caspase-1 were activated in the colon of mice in the DSS-induced group. In contrast, with the continuous administration of YTs during DSS induction, the expression of the three proteins was significantly decreased (*p* < 0.05), indicating that the NLRP3 inflammasome was inhibited by the YT continuous treatments. However, stopping the YT intervention during DSS induction, only LYT inhibits NLRP3 inflammasome activation. SYT and BYT do not show inhibition of NLRP3 activation (Figure 6a,b).

**Figure 7:**
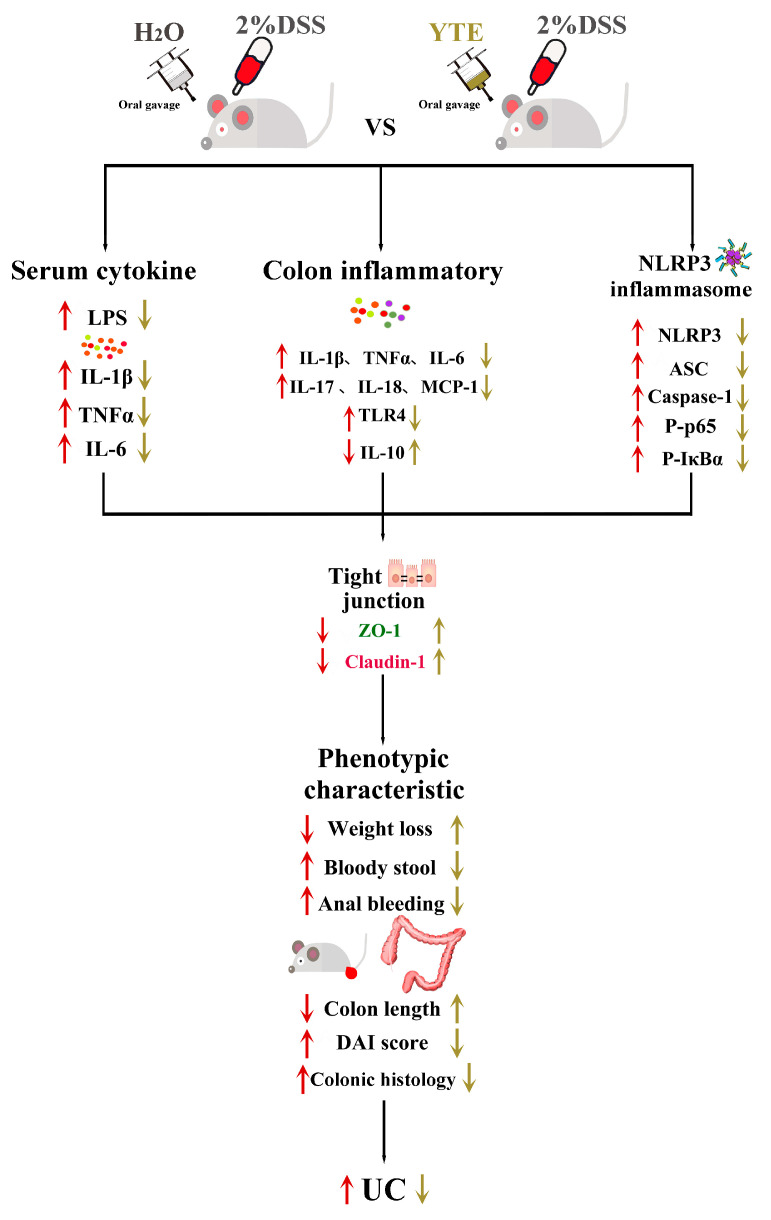
The schematic diagram for this study.

The NLRP3 inflammasome activation requires a specific initial signal pathway. In order to elucidate the impact of YTs on this pathway, we also analyzed the protein expression levels of NF-κB-related proteins in the colon of various groups of mice. As shown in Figure 6a,e–h, DSS induction significantly activated phosphorylated p65 (p-p65) and phosphorylated-IκBα (p-IκBα) expression, and with the continuous YT treatment during DSS induction, the increased expression of p-p65 and p-IκBα was prevented. However, the expression levels of total p65 and IκBα protein were not significantly different across the groups. It is noteworthy that pre-treatment of YTs for 4 weeks before DSS induction can significantly inhibit the activation of p-IκBα regardless of the YT treatments are stopped or continued during DSS induction. However, only continuous LYT intervention during DSS induction can effectively alleviate the increase in p-p65 expression level. These results also prove that LYT has better preventive and therapeutic effects on DSS-induced UC than BYT and SYT. The schematic diagram for this study is presented in Figure 7.

## 4. Discussion

UC is a chronic relapsing inflammatory bowel disease with an unknown etiology that only affects the colon and rectum. It is characterized by severe damage to the colonic mucosa and elevated concentrations of inflammatory cytokines. Although many drugs, including aminosalicylic acid, corticosteroids, immunomodulators, biologics, and antibiotics, can help alleviate UC symptoms, there is still no complete cure for UC so far. In addition, the accompanying side effects of the current drugs limit their clinical application, and the high cost of the drugs and the rising incidence of UC pose serious public health challenges worldwide. Therefore, there is an urgent need to find a new natural product to prevent and treat enteritis that is effective, safe and has few side effects.

Tea is one of the most popular non-alcoholic beverages due to its unique taste and wide range of health benefits. In Asian countries, tea intake has been found to be associated with a low incidence rate of inflammatory bowel disease. Researchers have found that moderate intake of epigallocatechin gallate (EGCG) can reduce the severity of diarrhea, prevent weight loss, and reduce macroscopic and histological inflammatory indicators in rodent models of colitis [31]. In addition, green tea polyphenols have been shown to treat DSS-induced colitis by upregulating nuclear transcription factor Nrf2, tight junction protein, and oxidative stress response protein, downregulating endoplasmic reticulum stress response, inflammatory response [32]. Furthermore, Fuzhuan tea crude extract significantly decreased TNF-α, IL-1β and IFN-γ inflammatory cytokines’ levels, and significantly reduced myeloperoxidase activity, nitric oxide, and malondialdehyde levels in colon tissue, thus improving DSS-induced colitis in mice [33]. Other studies have also reported that black tea extract alleviated DSS-induced colitis by blocking NF-κB signaling and cell apoptosis [34]. Pu-erh raw tea extract alleviates DSS-induced colitis in mice by restoring intestinal barrier function and maintaining gut microbiota homeostasis [35]. In addition, theabrownin from Pu-erh tea improves DSS-induced colitis by restoring gut homeostasis and inhibiting TLR2&4 signaling pathway [36]. The differences in the chemical composition of different types of tea may lead to differences in their in vitro and in vivo biological activities. Therefore, it would be very interesting to determine if YTs can also alleviate DSS-induced colitis in mice. Furthermore, a comparative investigation of the distinct effects of different type of YTs on UC has not yet been reported. In addition, the potential different effects of continuous and stopped consumption of YTs on UC during DSS induction are still unknown.

In this paper, the distinct effects of three types of YTs, including LYT, SYT and BYT, on DSS-induced UC were comparatively investigated. The results found that LYT has a significantly better preventive effect than SYT and BYT. This may be due to the fact that LYT is made from coarse mature leaves, which contain higher levels of polyphenols and other catechins [14], which may be beneficial to the preventive effects. Interestingly, our results show that continuous consumption of YTs has a more obvious reversal effect on UC than stopping consumption during DSS-induced UC. Pre-treatment of YTs for 4 weeks before DSS induction can alleviate the damage caused by DSS induction to a certain degree. This indicates that the consumption of YTs might be a good strategy to prevent IBD. In addition, continuous consumption of YTs at the same time as DSS induction significantly improves the DSS-induced UC, which suggests that YTs might exert a therapeutic effect on UC. 

The gut microbiota has been widely recognized in regulating intestinal homeostasis and IBD pathogenesis [37,38,39,40]. Dysfunctional gut microbiota in IBD includes an increase in facultative anaerobic bacteria and a decrease in obligate anaerobic producers of short-chain fatty acids (SCFA). A large number of clinical studies have also confirmed the beneficial effects of SCFA in regulating intestinal homeostasis and improving the onset of IBD [41,42,43]. Our research shows that three types of yellow tea have positive anti-inflammatory effects on the intestines and are also involved in repairing and improving intestinal functional integrity by inhibiting TLR4/NF-κB/NLRP3 inflammasome, which is supported by previous reports [22]. However, the effects of different type of YT consumptions on gut microbiota in DSS-induced UC mice and their exact mechanism in intestinal inflammation requires further investigation. This study also has certain limitations. The results rely on animal models and require further validation in clinical studies. In the future, we will continue to explore the long-term effects of regular consumption of YTs with clinical methods and investigate the mechanisms of YTs on other inflammatory pathways.

## Data Availability

The original contributions presented in the study are included in this article/Supplementary Materials; further inquiries can be directed to the corresponding authors.

## Supplementary Materials

The following supporting information can be downloaded at: https://www.mdpi.com/article/10.3390/foods13172843/s1, Table S1: DAI scoring standard; Table S2: Histological scoring criteria for colon sections; Table S3: The primers used for qRT-PCR analysis.

## References

[1] Ng S.C., Shi H.Y., Hamidi N., Underwood F.E., Tang W., Benchimol E.I., Panaccione R., Ghosh S., Wu J.C.Y., Chan F.K.L. (2017). Worldwide incidence and prevalence of inflammatory bowel disease in the 21st century: A systematic review of population-based studies. Lancet.

[2] Kobayashi T., Siegmund B., Le Berre C., Wei S.C., Ferrante M., Shen B., Bernstein C.N., Danese S., Peyrin-Biroulet L., Hibi T. (2020). Ulcerative colitis. Nat. Rev. Dis. Primers.

[3] König J., Wells J., Cani P.D., García-Ródenas C.L., MacDonald T., Mercenier A., Whyte J., Troost F., Brummer R.J. (2016). Human Intestinal Barrier Function in Health and Disease. Clin. Transl. Gastroenterol..

[4] Jena G., Trivedi P.P., Sandala B. (2012). Oxidative stress in ulcerative colitis: An old concept but a new concern. Free Radic. Res..

[5] Zou J., Liu C., Jiang S., Qian D., Duan J. (2021). Cross Talk between Gut Microbiota and Intestinal Mucosal Immunity in the Development of Ulcerative Colitis. Infect. Immun..

[6] Li Z., Zhang X., Liu C., Peng Q., Wu Y., Wen Y., Zheng R., Yan Q., Ma J. (2022). Macrophage-Biomimetic Nanoparticles Ameliorate Ulcerative Colitis through Reducing Inflammatory Factors Expression. J. Innate Immun..

[7] Danese S., Fiocchi C., Panés J. (2016). Drug development in IBD: From novel target identification to early clinical trials. Gut.

[8] Ordás I., Eckmann L., Talamini M., Baumgart D.C., Sandborn W.J. (2012). Ulcerative colitis. Lancet.

[9] Groschwitz K.R., Hogan S.P. (2009). Intestinal barrier function: Molecular regulation and disease pathogenesis. J. Allergy Clin. Immunol..

[10] He X., Wei Z., Wang J., Kou J., Liu W., Fu Y., Yang Z. (2016). Alpinetin attenuates inflammatory responses by suppressing TLR4 and NLRP3 signaling pathways in DSS-induced acute colitis. Sci. Rep..

[11] Schroder K., Zhou R., Tschopp J. (2010). The NLRP3 Inflammasome: A Sensor for Metabolic Danger?. Science.

[12] Villani A.C., Lemire M., Fortin G., Louis E., Silverberg M.S., Collette C., Baba N., Libioulle C., Belaiche J., Bitton A. (2009). Common variants in the NLRP3 region contribute to Crohn’s disease susceptibility. Nat. Genet..

[13] Bauer C., Duewell P., Mayer C., Lehr H.A., Fitzgerald K.A., Dauer M., Tschopp J., Endres S., Latz E., Schnurr M. (2010). Colitis induced in mice with dextran sulfate sodium (DSS) is mediated by the NLRP3 inflammasome. Gut.

[14] Feng X., Yang S., Pan Y., Zhou S., Ma S., Ou C., Fan F., Gong S., Chen P., Chu Q. (2023). Yellow tea: More than turning green leaves to yellow. Crit. Rev. Food Sci. Nutr..

[15] Guo X., Ho C.T., Schwab W., Song C., Wan X. (2019). Aroma compositions of large-leaf yellow tea and potential effect of theanine on volatile formation in tea. Food Chem..

[16] Shin H., Seo D.-H., Seo J., Lamothe L.M., Yoo S.-H., Lee B.-H. (2019). Optimization of in vitro carbohydrate digestion by mammalian mucosal α-glucosidases and its applications to hydrolyze the various sources of starches. Food Hydrocolloids.

[17] Teng Y., Li D., Guruvaiah P., Xu N., Xie Z. (2018). Dietary Supplement of Large Yellow Tea Ameliorates Metabolic Syndrome and Attenuates Hepatic Steatosis in db/db Mice. Nutrients.

[18] Qian S.W., Tang Y., Li X., Liu Y., Zhang Y.Y., Huang H.Y., Xue R.D., Yu H.Y., Guo L., Gao H.D. (2013). BMP4-mediated brown fat-like changes in white adipose tissue alter glucose and energy homeostasis. Proc. Natl. Acad. Sci. USA.

[19] Xu N., Chu J., Dong R., Lu F., Zhang X., Wang M., Shen Y., Xie Z., Ho C.T., Yang C.S. (2021). Yellow Tea Stimulates Thermogenesis in Mice through Heterogeneous Browning of Adipose Tissues. Mol. Nutr. Food Res..

[20] Shen L., Liu L., Ji H.-F. (2016). Alzheimer’s Disease Histological and Behavioral Manifestations in Transgenic Mice Correlate with Specific Gut Microbiome State. J. Alzheimer’s Dis..

[21] Marques F.Z., Mackay C.R., Kaye D.M. (2018). Beyond gut feelings: How the gut microbiota regulates blood pressure. Nat. Rev. Cardiol..

[22] Liu H., Chen R., Wen S., Li Q., Lai X., Zhang Z., Sun L., Sun S., Cao F. (2023). Tea (*Camellia sinensis*) ameliorates DSS-induced colitis and liver injury by inhibiting TLR4/NF-κB/NLRP3 inflammasome in mice. Biomed. Pharmacother..

[23] Li B.Y., Mao Q.Q., Gan R.Y., Cao S.Y., Xu X.Y., Luo M., Li H.Y., Li H.B. (2021). Protective effects of tea extracts against alcoholic fatty liver disease in mice via modulating cytochrome P450 2E1 expression and ameliorating oxidative damage. Food Sci. Nutr..

[24] Lai X., Wang X., Wen S., Sun L., Chen R., Zhang Z., Li Q., Cao J., Lai Z., Li Z. (2022). Six Types of Tea Reduce Acute Alcoholism in Mice by Enhancing Ethanol Metabolism, Suppressing Oxidative Stress and Inflammation. Front. Nutr..

[25] Cooper H.S., Murthy S.N., Shah R.S., Sedergran D.J. (1993). Clinicopathologic study of dextran sulfate sodium experimental murine colitis. Lab. Investig..

[26] Wu G., Gu W., Cheng H., Guo H., Li D., Xie Z. (2022). Huangshan Maofeng Green Tea Extracts Prevent Obesity-Associated Metabolic Disorders by Maintaining Homeostasis of Gut Microbiota and Hepatic Lipid Classes in Leptin Receptor Knockout Rats. Foods.

[27] Yun H.F., Liu R., Han D., Zhao X., Guo J.W., Yan F.J., Zhang C., Sun H.W., Liang G.Q., Zhang G.X. (2020). Pingkui Enema Alleviates TNBS-Induced Ulcerative Colitis by Regulation of Inflammatory Factors, Gut Bifidobacterium, and Intestinal Mucosal Barrier in Rats. Evid. Based Complement. Alternat Med..

[28] Xing D., Li T., Ma G., Ruan H., Gao L., Xia T. (2021). Transcriptome-Wide Analysis and Functional Verification of RING-Type Ubiquitin Ligase Involved in Tea Plant Stress Resistance. Front. Plant Sci..

[29] Xie Z., Su W., Liu S., Zhao G., Esser K., Schroder E.A., Lefta M., Stauss H.M., Guo Z., Gong M.C. (2015). Smooth-muscle BMAL1 participates in blood pressure circadian rhythm regulation. J. Clin. Investig..

[30] Landy J., Ronde E., English N., Clark S.K., Hart A.L., Knight S.C., Ciclitira P.J., Al-Hassi H.O. (2016). Tight junctions in inflammatory bowel diseases and inflammatory bowel disease associated colorectal cancer. World J. Gastroenterol..

[31] Abboud P.A., Hake P.W., Burroughs T.J., Odoms K., O’Connor M., Mangeshkar P., Wong H.R., Zingarelli B. (2008). Therapeutic effect of epigallocatechin-3-gallate in a mouse model of colitis. Eur. J. Pharmacol..

[32] Wu Z.H., Huang S.M., Li T.T., Li N., Han D.D., Zhang B., Xu Z.J.Z., Zhang S.Y., Pang J.M., Wang S.L. (2021). Gut microbiota from green tea polyphenol-dosed mice improves intestinal epithelial homeostasis and ameliorates experimental colitis. Microbiome.

[33] Liu B., Yang T., Zeng L., Shi L., Li Y., Xia Z., Xia X., Lin Q., Luo F. (2016). Crude extract of Fuzhuan brick tea ameliorates DSS-induced colitis in mice. Int. J. Food Sci. Technol..

[34] Song Y.-A., Park Y.-L., Kim K.-Y., Chung C.-Y., Lee G.-H., Cho D.-H., Ki H.-S., Park K.-J., Cho S.-B., Lee W.-S. (2011). Black tea extract prevents lipopolysaccharide-induced NF-κB signaling and attenuates dextran sulfate sodium-induced experimental colitis. BMC Complem. Altern. Med..

[35] Zhou S., Yang J., Pan Y., Feng X., Hu H., Ma S., Ou C., Fan F., Gong S., Wang Y. (2023). Pu’ er raw tea extract alleviates DSS-induced colitis in mice by restoring intestinal barrier function and maintaining gut microbiota homeostasis. Food Biosci..

[36] Zhao L., Zhao C., Miao Y., Lei S., Li Y., Gong J., Peng C. (2024). Theabrownin from Pu-erh tea improves DSS-induced colitis via restoring gut homeostasis and inhibiting TLR2&4 signaling pathway. Phytomedicine.

[37] Yinping P., Zhang H., Li M., He T., Guo S., Zhu L., Tan J., Wang B. (2024). Novel approaches in IBD therapy: Targeting the gut microbiota-bile acid axis. Gut Microbes.

[38] Liu Y., Bai X., Wu H., Duan Z., Zhu C., Fu R., Fan D. (2024). Ginsenoside CK Alleviates DSS-Induced IBD in Mice by Regulating Tryptophan Metabolism and Activating Aryl Hydrocarbon Receptor via Gut Microbiota Modulation. J. Agric. Food Chem..

[39] Sugihara K., Kamada N. (2024). Metabolic network of the gut microbiota in inflammatory bowel disease. Inflamm. Regen..

[40] Sun M.M., Wu W., Liu Z.J., Cong Y.Z. (2017). Microbiota metabolite short chain fatty acids, GPCR, and inflammatory bowel diseases. J. Gastroenterol..

[41] Deleu S., Machiels K., Raes J., Verbeke K., Vermeire S. (2021). Short chain fatty acids and its producing organisms: An overlooked therapy for IBD?. EBioMedicine.

[42] Akhtar M., Chen Y., Ma Z.Y., Zhang X.L., Shi D.S., Khan J.A., Liu H.Z. (2022). Gut microbiota-derived short chain fatty acids are potential mediators in gut inflammation. Anim. Nutr..

[43] Machiels K., Joossens M., Sabino J., De Preter V., Arijs I., Eeckhaut V., Ballet V., Claes K., Van Immerseel F., Verbeke K. (2014). A decrease of the butyrate-producing species Roseburia hominis and Faecalibacteriumprausnitzii defines dysbiosis in patients with ulcerative colitis. Gut.

